# Ancient Methods Deliver a Current Message

**DOI:** 10.3201/eid2705.AC2705

**Published:** 2021-05

**Authors:** Byron Breedlove

**Affiliations:** Centers for Disease Control and Prevention, Atlanta, Georgia, USA

**Keywords:** art science connection, emerging infectious diseases, art and medicine, about the cover, ancient methods deliver a current message, handwashing is important, 2020, Ambika Devi, Madhubani, ancient methods deliver a current message, coronavirus disease, COVID-19, severe acute respiratory syndrome coronavirus 2, SARS-CoV-2, coronaviruses, viruses, respiratory infections, handwashing, masks, social distancing, pandemic, World Health Organization, WHO, India

**Figure:**
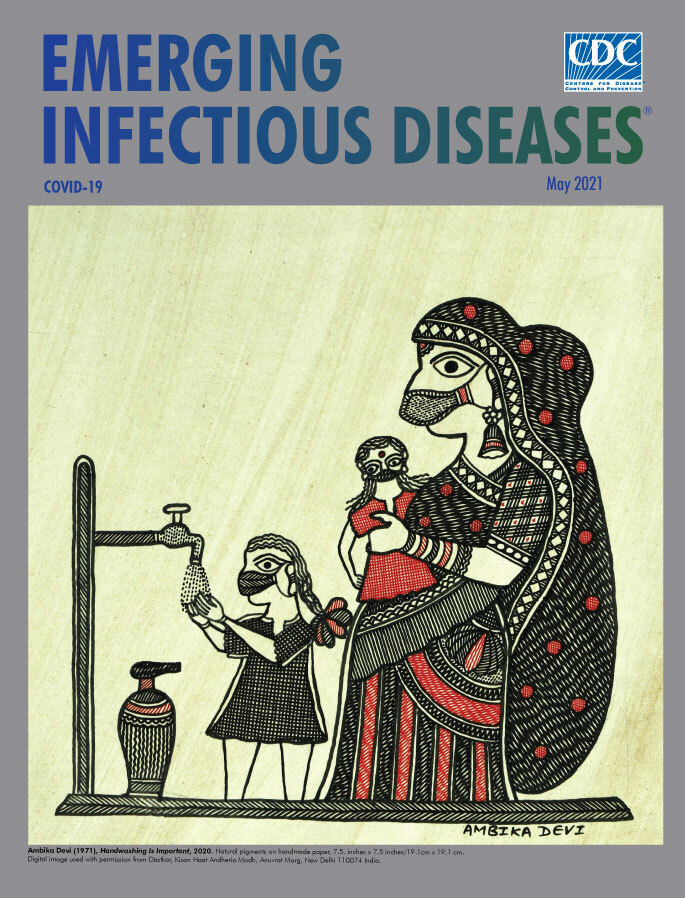
**Ambika Devi (1971−), *Handwashing Is Important*, 2020.** Natural pigments on handmade paper, 7.5. in x 7.5 in/19.1 cm x 19.1 cm. Digital image used with permission from Dastkar, Kisan Haat Andheria Modh, Anuvrat Marg, New Delhi 110074, India.

In March 2020, the World Health Organization classified the COVID-19 outbreak as a pandemic. According to data from the WHO Coronavirus (COVID-19) Dashboard (April 12, 2021), ≈137,000,000 confirmed cases of COVID-19, including ≈2,930,000 confirmed deaths, have been documented in 223 countries. Actual numbers of cases and deaths are larger because of challenges with testing and determining causes of death.

The UN Educational, Scientific and Cultural Organization (UNESCO) notes that the culture sector, which includes more than 30 million people globally, has been hit hard by the coronavirus pandemic. Nonetheless, artists everywhere have responded to the pandemic; many are not only depicting their experiences and circumstances but are also promoting public health practices that can aid in reducing the spread of COVID-19, such as washing hands and wearing masks.

Journalist Sudha G. Tilak writes that “India's folk artists have long used traditional art for social messaging” and that since the start of the COVID-19 pandemic, “a number of those artists have released works that reinforce and promote those basic public health practices.” Tilak also quotes Laila Tyabji, chairperson of Dastkar, a private nonprofit organization that supports traditional craftspeople in India, who said, “Though many fear the impact of COVID-19 may be the end of craftspeople, it is their creativity and resilience that could save them.” For context, WHO Coronavirus Dashboard data (April 12, 2021) indicate that India ranks second among the world’s countries in cumulative total of confirmed cases of COVID-19 and fourth in confirmed deaths.

This month’s cover image is a modern example of an ancient art genre known as Madhubani by Indian artist Ambika Devi, from the village of Rashidpur in the Madhubani district of Bihar, an ancient city now divided between India and Nepal. Devi has been practicing Madhubani art since she was 12 years old. She learned its traditional techniques from her mother, Leela Devi, who in turn, learned them from her mother. Devi, considered a master of this art form, has won multiple awards for her work―including the President of India's National Handicrafts Award for her contributions to art (2009), the Crafts Council of India’s Kamala Award for Excellence in Craftsmanship (2018), the Sanmaan Award for Excellence in Handicrafts by the Crafts Council of Telangana (2019), and Jasthi Ramaiah Award for Excellence in Craft by the Craft Council of Telangana (2021).

For centuries, Madhubani has been practiced in the northern corner of India and adjoining parts of Terai in southern Nepal. Madhubani artisans use natural dyes and pigments created from various leaves, berries, and tree bark, fresh turmeric, soot, and even cow dung. Using twigs, brushes, nib pens, and their fingers, the artists create colorful intricate works filled with complex geometrical patterns on paper, canvas, fabric, walls, and other surfaces.

In this work, an example of the Katchni (line style) of Madhubani, Devi limits her palette to red and black pigments on a light background. She shows a mother and children washing hands by a pump―an everyday scene that could be from many village squares. Devi’s initial steps involve painstakingly drawing complex patterns on a blank surface. Next, she meticulously fills in the shapes and forms with pigments or dyes and uses double lines to set off the foreground from the background and to add depth. The various stripes, textures, fringes, brocades, jewelry, and other details of the mother’s clothing create a kaleidoscopic array of shapes that dominates the painting. An older child is shown washing her hands near her watchful mother who holds a younger child. All three are wearing face masks as protection from COVID-19, and Devi’s use of an ancient craft to depict this very modern behavior does not seem in the least incongruous.

Since the start of the COVID-19 pandemic, people everywhere have been adjusting to social distancing and mask wearing. Hospitals are being overwhelmed by admissions and healthcare professionals working to exhaustion. The evidence confirming that wearing masks is an effective nonpharmacologic way to reduce the spread of COVID-19 continues to grow. Devi’s painting relies on traditional artistic methods to provide a glimpse of life in her village during this pandemic and to deliver a contemporary message, gently reminding viewers of ways to protect their health and the health of others.
